# Intravenous Thrombolysis After Dabigatran Reversal by Idarucizumab: A Systematic Review of the Literature

**DOI:** 10.3389/fneur.2021.666086

**Published:** 2021-06-03

**Authors:** Senta Frol, Dimitrios Sagris, Janja Pretnar Oblak, Mišo Šabovič, George Ntaios

**Affiliations:** ^1^Department of Vascular Neurology, University Clinical Centre Ljubljana, Faculty of Medicine, University of Ljubljana, Ljubljana, Slovenia; ^2^Department of Internal Medicine, Faculty of Medicine, School of Health Sciences, University of Thessaly, Larissa, Greece; ^3^Department of Vascular Disorders, University Clinical Centre Ljubljana, Ljubljana, Slovenia

**Keywords:** dabigatran, idarucizumab, ischemic stroke, intravenous thrombolysis, outcome

## Abstract

**Background and Purpose:** Idarucizumab achieves instant reversal of anticoagulation and enables intravenous thrombolysis (IVT) in dabigatran-treated acute ischemic stroke (AIS) patients. AIS in dabigatran-treated patients is a rare event, therefore the experience is limited. A review of all published cases was performed to evaluate the safety and effectiveness of this therapeutic strategy.

**Methods:** We searched PubMed and Scopus for all published cases of IVT after reversal with idarucizumab in dabigatran-treated AIS patients. The outcomes were safety assessed by hemorhagic transformation (HT), symptomatic intracranial hemorrhage (SICH) and death, and efficacy assessed by National Institutes of Health Stroke Scale (NIHSS) reduction.

**Results:** We identified 251 AIS patients (39,9% females) with an average age of 74 years. HT, SICH, and death were reported in 19 (7.6%), 9 (3.6%), and 21 (8.4%) patients, respectively. Patients experiencing HT presented with more severe strokes (median NIHSS on admission: 21 vs. 8, *p* < 0.001; OR: 1.12, 95% CI: 1.05–1.20). After IVT there was a significant NIHSS reduction of 6 points (IQR:3–10, *p* < 0.001) post-stroke and linear regression revealed a correlation of admission NIHSS to NIHSS reduction (*p* < 0.001).

**Conclusions:** In this systematic review of all published cases of IVT in dabigatran-treated AIS patients after reversal with idarucizumab the rates of HT, SICH and mortality, as well as NIHSS reduction, were comparable with previous studies in non-anticoagulated patients. This provides reassuring evidence about the safety and efficacy of this therapeutic strategy.

## Introduction

Idarucizumab is a specific reversal agent for dabigatran, which achieves reversal of anticoagulation within a few minutes after application without thrombotic or other side-effects (1, 2). The use of idarucizumab is indicated in dabigatran-treated patients with life-threatening and uncontrolled bleeding and those who need urgent surgery or intervention (2, 3). The updated 2018 European Heart Rhythm Association guidelines and other expert panels recommend reversal with idarucizumab for dabigatran-treated patients with acute ischemic stroke (AIS) if they are eligible candidates for intravenous thrombolysis (IVT) (4, 5).

AIS in dabigatran-treated patients is a rare event (6, 7), with yearly incidences of 0.9% for the standard dose and 1.3% for the reduced dose (8), therefore the experience is expected to be limited. The first case reports describing IVT in dabigatran-treated AIS patients who received idarucizumab were published in 2014. A previous systematic review reported lower rates of symptomatic intracranial hemorrhage (SICH) and death as well as higher rates of favorable outcome after IVT among 44 dabigatran/idarucizumab treated patients compared to 108 dabigatran treated patients without idarucizumab reversal (9). During the recent years the number of case reports and case series grew, providing more data on patients treated with IVT after dabigatran reversal with idarucizumab (1, 10–57). It is expected that the use of anticoagulants, including dabigatran, will continue to progressively rise.

The aim of the present systematic review was to summarize all published cases of dabigatran-treated patients with AIS who were treated with IVT after reversal with idarucizumab. This could be very useful to stroke physicians when treating dabigatran-treated patients with AIS.

## Methodology

### Search Strategy and Inclusion Criteria

We searched PubMed and Scopus until 27/10/2020 for studies reporting AIS patients treated with IVT after dabigatran reversal with idarucizumab using the terms: “idarucizumab” or “reversal” or “dabigatran” and “thrombolysis” or “tpa” or “alteplase” or “tissue plasminogen activator” or “tenecteplase” and “stroke” or “cerebrovascular.” The search was limited from 01/01/2013 to 27/10/2020, since the term “idarucizumab” was first reported during 2014. In addition, we searched the references of related letters, reviews and editorials to identify other potentially eligible studies. We also contacted experts in the field to identify potential missed studies. To be eligible for the present analysis, the studies had to be published full-text articles in English language, providing data about the characteristics and outcomes of interest in patients treated with IVT after dabigatran reversal with idarucizumab. This work was reported according to the PRISMA statement (58) and registered in PROSPERO (CRD42020207198).

### Outcomes and Data Extraction

The outcomes assessed were HT, SICH (both as defined in the included case reports/series), death and the change in the National Institutes of Health Stroke Scale (NIHSS). A follow-up head computed tomography (CT) scan or magnetic resonance imaging (MRI) was obtained 24 h after IVT in each included case. Additional CT (or MRI) was obtained only if neurological deterioration occurred. HT was defined as the presence of any blood in the ischemic region obtained on the control CT. Precise definitions of SICH are presented in Supplementary Table 1 if applicable.

Eligible studies were assessed independently by the first two authors. Individual patient data were extracted from single-case reports and case series whenever possible using prespecified forms. Quality assessment of the included case reports and case series performed with a tool made to evaluate the methodological quality of case reports and case series (59).

### Methodology and Statistical Analysis

Patient characteristics and outcomes were described based on two different prespecified approaches: individual patient data from single case-reports or case-series that provided individual patient data, were pooled together to form a unified group of patients. Case-series that provided aggregate data rather than individual patient data, were reported separately. Discrete variables reported using proportions and continuous variables as means with standard deviations (SD) or medians with interquartile range (IQR). Univariate comparisons were performed from non-parametric Kruskall Wallis test for continuous variable's or Pearson's chi-squared test for categorical variables. The non-parametrical Wilcoxon signed-rank test was used to compare admission NIHSS to NIHSS at 24 h or at discharge. We performed logistic regression analysis to evaluate the association between admission NIHSS and the risk of hemorrhagic transformation and death. Estimates are provided as odds ratio (OR) and 95% confidence intervals (CI). The level of statistical significance was set at 5%. Statistical analyses were performed with the StataCorp. 2015. *Stata Statistical Software: Release 14*. College Station, TX: StataCorp LP.

## Results

**Figure 1:**
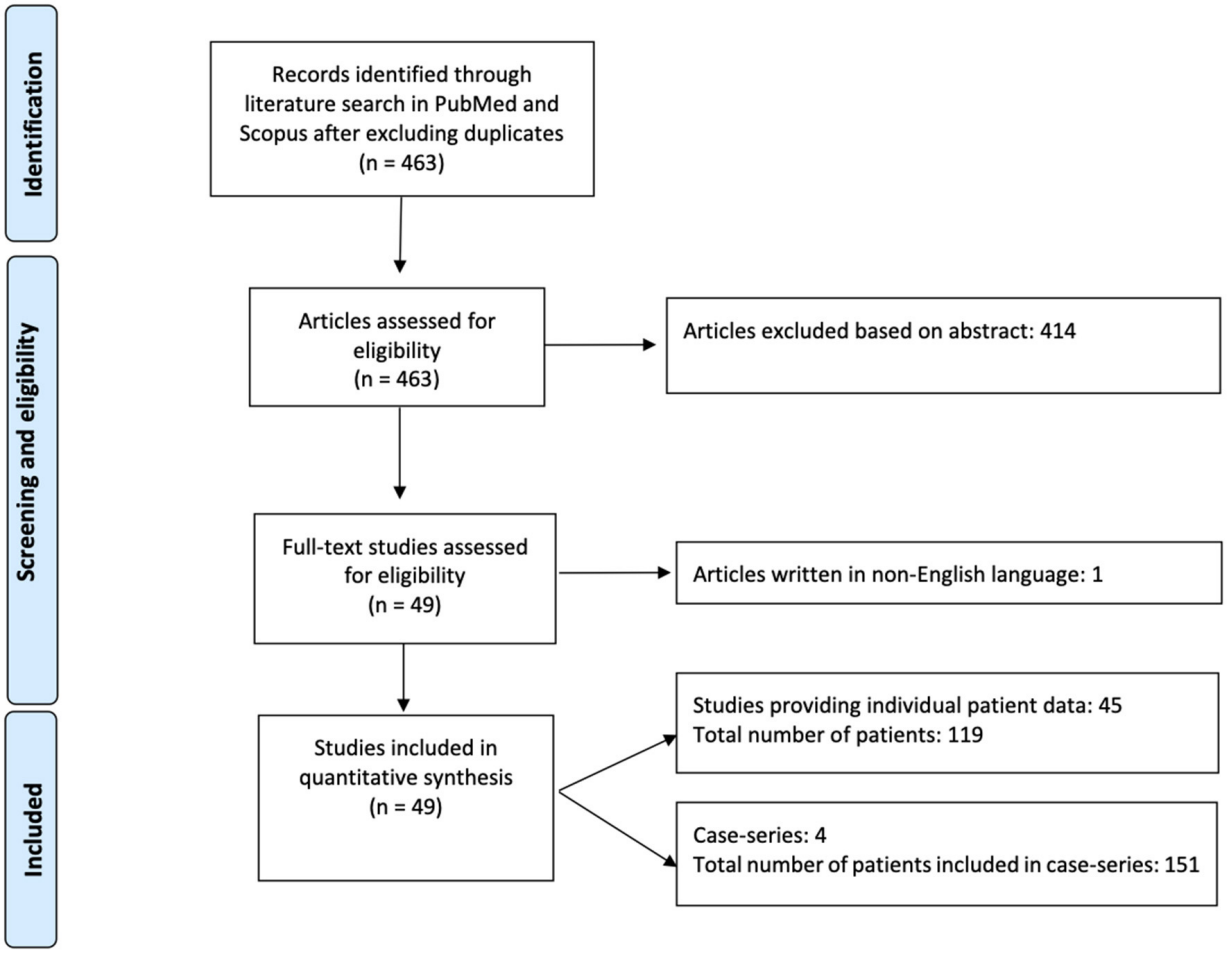
Flow diagram of studies identified, screened, and included in the analysis.

Among 463 articles identified from the initial literature search, 49 studies including 251 patients were eligible and included in the analysis (1, 13–57, 60–63) (Figure 1). Forty-five studies provided individual patient data of 119 AIS patients (13–23, 25, 27–36, 38–57, 60–63), whereas four case-series reported results on overall 151 patients (1, 24, 26, 37).

Data about 19 patients included in a paper from Kermer et al. (13) were published later again in a paper from Kermer et al. (1). Therefore, in the overall weighted analysis of individual patient data and case-series, we did not include the first study (13).

The case-series which reported individual patient data was used in the individual patient analysis (13) and the case-series which reported overall results was used in the aggregate patient analysis (1).

The baseline characteristics of the included studies and the quality assessment can be found in Supplementary File (Supplementary Tables 1, 2).

The average age was 74 years and 97 (38.6%) were females. Overall, 19 patients (7.6%) experienced a HT and among them 9 (3.6%) were complicated with SICH. Twenty-one patients (8.4%) died.

### Individual Patient Data Subanalysis

Among 119 patients included in single case reports or case-series that provided individual patient data, the mean age was 73.8 ± 11.3 years and 50 (42%) patients were females. The median stroke-to-needle time was 155 (IQR: 120–210) min and the median NIHSS at admission was 10 (IQR: 6–16). Thirteen patients (10.9%) were treated with endovascular thrombectomy (EVT) additionally to IVT. The baseline characteristics of patients are summarized in Table 1.

**Table 1:** Baseline characteristics of thrombolysed patients treated with idarucizumab.

**References**	**Patients no**.	**Age, mean ± SD**	**Female gender, *n* (%)**	**aPTT (sec), mean ± SD**	**Dabigatran dosage**	**Time since last dabigatr an dose (mins)**	**Stroke to needle time (min)**	**NIHSS on admission, median (IQR)**	**IV-rtPA**	**EVT**	**Antithrombotic at discharge**	**Time of anticoagulation restart (days)**
Kermer et al. (1)	80	75.9 ± 10.7	29 (36.3)	42.7 ± 15.1	150 mg, 32 110 mg, 48	-	-	9 (IQR na)	0.9 mg/Kg Alteplase	6 (7.5)	dabigatran, 39 apixaban, 11 edoxaban, 1 rivaroxaban, 1 phenprocoumon, 3	1–10
Barber et al. (26)	51	73.3 ± 12.2	14 (27.5)	-	-	-	163 (121–123)	8 (5–17)	-	8 (15.7)	-	-
Šanák et al. (24)	13	70.0 ± 9. 1	5 (38.5)	38.1 ± 27.8	150 mg, 8 110 mg, 5	291 ± 235	158 ± na^¶^	7 (3–23)^*^	0.9 mg/Kg Alteplase	0	Dabigatran, 7 apixaban, 3	5–120
Küpper et al. (37)	7	-	-	-	-	-	-	-	0.9 mg/Kg Alteplase	4 (57.1)	-	
Pooled data from 119 case reports (including 19 cases from Kermer et al.)^*^	119	73.8 ± 11.3	50 (42)	44.4 ± 18.6, 85 obs	110 mg, 54 150 mg, 35 75 mg, 1 90 obs	441 ± 366, 43 obs	155 (120–210), 66 obs	10 (6–16) 116obs	0.6 mg/Kg: 6 0.7 mg/Kg: 3 0.9 mg/Kg: 89 tenecteplase:3 (101 obs)	16 (13.5)	Warfarin, 6 Apixaban, 4 Dabigatran, 43 Rivaroxaban, 4 ASA+warfarin, 1 ASA+Dabigatran, 2 ASA 1 (62 obs)	3 (2–7) 38 obs
Weighted pooled data of 251 patients; observations in each variable	251	74; 244	97 (38.6); 243	43.2; 158		407; 56	166; 129	9; 240		30 (12)	Dabigatran, 77 apixaban, 16 Edoxaban, 1 Rivaroxaban, 3 ASA+warfarin, 1 ASA+Dabigatran, 2 ASA 1; 136	

*SD, standard deviation; aPTT, activated partial thromboplastin time; NIHSS, National Institute of Health Stroke Scale; EVT, Endovascular thrombectomy; IV-rtPA, intravenous recombinant tissue plasminogen activator; EVT, intravenous thrombolysis*.

* *Individual data retrieved from case reports. All case reports can be found in Supplementary Material*.

HT was reported in 15 patients (12.6%) and among them, six patients (5%) experienced SICH. Patients experiencing a HT presented with more severe strokes (median NIHSS on admission: 21 vs. 8, *p* < 0.001; OR: 1.12, 95% CI: 1.05–1.20).

**Figure 2:**
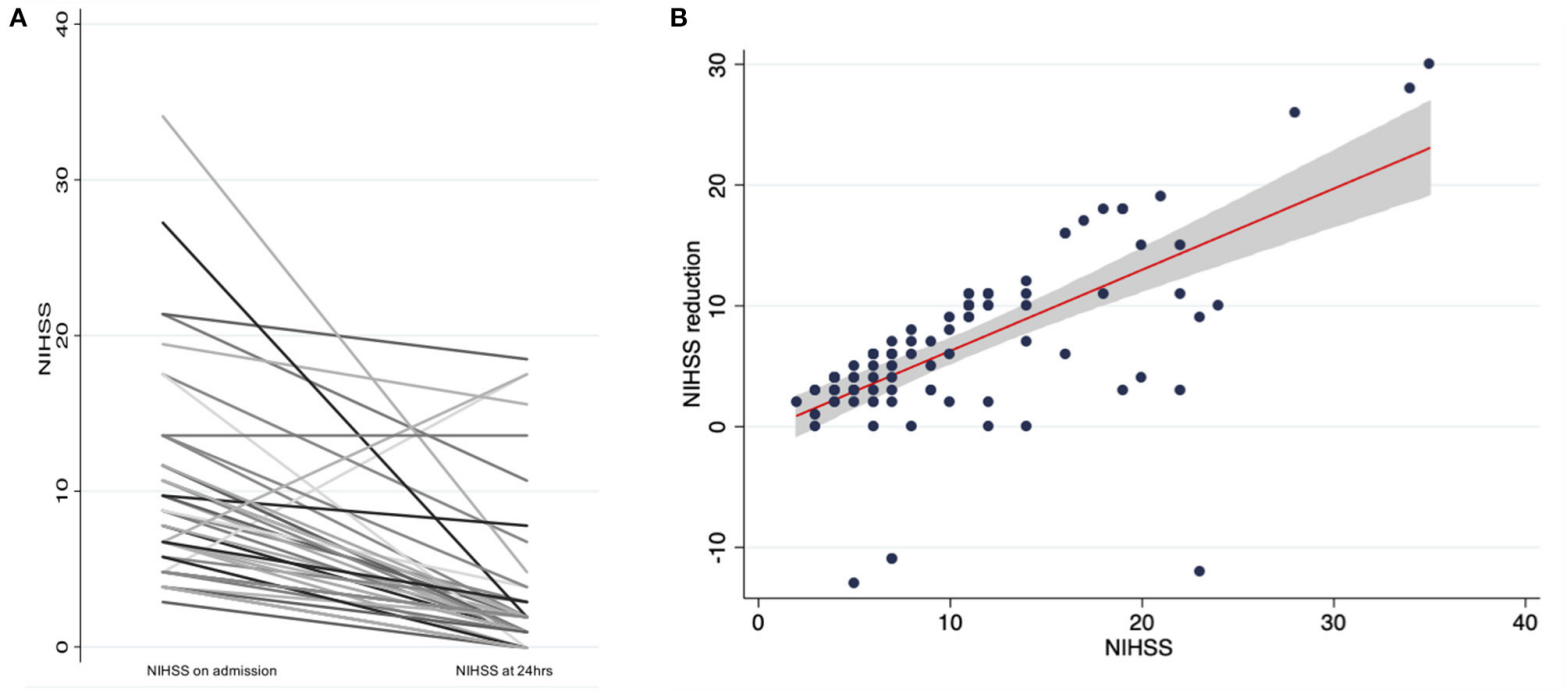
**(A)** NIHSS change in thrombolysed patients after dabigatran reversal with idarucizumab, **(B)** Linear regression analysis and 95% confidence interval of NIHSS reduction based on admission NIHSS.

Patients who were treated with IVT after idarucizumab reversal of dabigatran had a significant median NIHSS reduction of six (IQR: 3–10, Wilcoxon sign-ranked test, *p* < 0.001; Figure 2A). In the sub analysis a linear correlation between initial stroke severity (NIHSS at admission) and the outcome (evaluated as NIHSS improvement), controlling for age and gender was found (*p* < 0.001) (Figure 2B). Death was reported in 10 patients (8.4%) who presented with significantly higher admission NIHSS compared to stroke survivors (23 vs. 9, OR: 1.21, 95% CI: 1.09–1.33) (Table 2).

**Table 2:** Outcomes of patients thrombolysed after dabigatran reversal with idarucizumab.

**References**	**Patients no**.	**NIHSS after IVT, median (IQR)**	**NiHSS reduction**	**Hemorrhagic transformation (%)**	**SICH (%)**	**Death (%)**
Kermer et al. (1)	80	2 (IQR na)	-	0	0	3 (3.8)
Barber et al. (26)	51	3 (1.5–5.5)	-	2 (3.9)	2 (3.9)	3 (5.9)
Šanák et al. (24)	13	3 (0–27)^*^	-	2 (15.4)	1 (7.7)	3 (23.1)
Küpper et al. (37)	7	-	-	0	0	2 (28.6)
Pooled data from 119 case reports (including 19 cases from Kermer et al.)	119	2 (1–4) (105 obs)	6 (3–10)	15 (12.6)	6 (5)	10 (8.4)
Weighted pooled data of 251 patients; observations in each variable		2.3; 231		19 (7.6)	9 (3.6)	21 (8.4)

*NIHSS, National Institute of Health Stroke Scale; IVT, intravenous thrombolysis; IQR, interquartile range; SICH, symptomatic intracranial hemorrhage*

* * Individual data retrieved from case reports. All case reports can be found in Supplementary Material*.

### Aggregate Patient Data Subanalysis

Four studies reported aggregate data for 151 patients who were treated with IVT after dabigatran reversal with idarucizumab (1, 24, 26, 37). The baseline characteristics of each case series are summarized in Table 1. Hemorrhagic transformation was reported in 4 patients (2.7%) and only one patient (0.7%) was complicated with SICH. Death was reported in 11 patients (7.2%) (Table 2).

## Discussion

In a systematic review of all published cases of IVT in idarucizumab/dabigatran-treated AIS patients published up until the end of October, 2020 (1, 13–57, 60–63), we confirmed the safety and efficacy of this treatment. IVT after dabigatran reversal with idarucizumab resulted in similar rate of HT, SICH and death, as well as similar reduction of NIHSS compared with previous studies in non-anticoagulated patients (64, 65). HT and death occurred in patients presenting with severe AIS. Importantly, effectiveness was the same regardless of stroke severity and age.

We have performed a comprehensive review of all published cases of IVT (up until October, 2020) after dabigatran reversal in patients with AIS with the aim to explore the safety and efficacy of idarucizumab reversal. Forty-seven studies were eligible and included in the analysis, with 251 AIS patients, who have been treated with IVT after reversal. So far the safety and efficacy of IVT after dabigatran reversal with idarucizumab has not been evaluated in large studies. In 2017 a small meta-analysis of all published cases (*n* = 21) with the review was done by Pikija et al. (15) suggesting good efficacy and safety of idarucizumab reversal. In 2020, so far the largest cohort of dabigatran-treated patients receiving IVT after reversal with idarucizumab (*n* = 80) in Germany was published (1). A significant clinical improvement was found in 78% of patients, neither bleeding nor thrombotic complications were associated with IVT after idarucizumab treatment. Thus, meta-analysis of all published cases from different regions and including patients of all ethnicities could be clinically very relevant and helpful to clinicians all around the world when treating dabigatran-treated patients with AIS.

Our analysis of safety revealed HT in 19 (7.6%) of patients and SICH in only 9 (3.6%) patients. Twenty-one patients (8.4%) died. The results are in line with the safety observed in studies of IVT in patients without previous anticoagulation (64, 65). Thus, pretreatment with dabigatran and idarucizumab at stroke onset and IVT is not accompanied with an increased risk of hemorrhage or death.

A significant median NIHSS reduction of six points was achieved, confirming the efficacy of this treatment being at least similar, if not better, than obtained in non-anticoagulated patients (64, 65). IVT after idarucizumab reversal was effective regardless of stroke severity and age. In the sub analysis a linear correlation between initial stroke severity and the outcome (adjusted for age and gender) was found.

In our analysis we recorded a high success rate of IVT after reversal in dabigatran-treated patients which was also described in two previous smaller analyses (1, 15). Although it is highly speculative this was previously presumed to be a possible consequence of higher sensitivity of thrombi in dabigatran-treated patients to lysis (11). Indeed, *in vitro* studies showed the ability of dabigatran to enhance the susceptibility of plasma clots to rt-PA-induced lysis by reducing thrombin-activatable fibrinolysis inhibitor (TAFI) activation and by altering the clot structure (66).

Our review has several limitations. Missing and uncomplete data on times of stroke onset to IVT and on discharge NIHSS, discharge mRS and mRS at 3 months in big cohorts are the main limitation and did not allow the analysis of these parameters. The lack of comparator group and the fact that efficacy and safety could not be directly compared with the results of alteplase trials are additional limitations. Clearly, further studies are needed to definitively confirm the findings of our meta-analysis. Registry such as Registry of Acute Stroke Under Novel Oral Anticoagulants-Prime (RASUNOA-Prime) (67), where patients are systematically followed-up for management, clinical course and outcome, will provide detailed insights into outcome of these patients.

In conclusion, our meta-analysis of all published cases of dabigatran-treated patients, who received idarucizumab as a reversal agent, confirmed the safety and efficacy of treatment in all AIS patients, treated with dabigatran, receiving IVT after reversal, regardless of age and stroke severity on admission. Nonetheless, admission NIHSS score appeared to be an independent predictor of mortality.

## Data Availability Statement

The original contributions presented in the study are included in the article/Supplementary Material, further inquiries can be directed to the corresponding author/s.

## Author Contributions

SF and DS: study design, data acquisition, statistical analysis and interpretation, and manuscript preparation. JP and MŠ: data acquisition, statistical analysis and interpretation, and critical revision of the manuscript. GN: study concept and design, statistical analysis and interpretation, manuscript preparation, and study supervision. All authors contributed to the article and approved the submitted version.

## Conflict of Interest

In the past 2 years, SF, MŠ, and JP have received honoraria for oral presentations from Bayer, Boehringer Ingelheim and Pfizer. GN reports Speaker fees/Advisory Boards/Research support from Abbott; Amgen; Bayer; Boehringer-Ingelheim; Elpen; Pfizer outside the submitted work. The remaining author declares that the research was conducted in the absence of any commercial or financial relationships that could be construed as a potential conflict of interest.

## Abbreviations

AIS: acute ischemic stroke
IVT: intravenous thrombolysis
SICH: symptomatic intracranial hemorrhage
HT: hemorrhagic transformation
NIHSS: National Institutes of Health Stroke Scale
OR: odds ratio
CI: confidence interval.
