# Supplementary material for: Intravenous Thrombolysis After Dabigatran Reversal by Idarucizumab: A Systematic Review of the Literature
Source: Front Neurol. 2021 Jun 3;12:666086. doi: 10.3389/fneur.2021.666086 (PMC8209294; doi:10.3389/fneur.2021.666086)
Supplement: Supplementary file 1 [file Data_Sheet_1.PDF]

Supplementary material

Table 1: Basic characteristics of the included case reports and case series

Study	Study type	Ethnicity	Patients included	Age	Female patients	NIHSS	aPTT (sec)	Dabigatran dosage	Stroke to idarucizumab (min)	Alteplase dosage (mg/Kg)	EVT	Hemorrhagic transformation	Death	SICH	SICH definition	INR	Hemo	TT
**Fang, C. W., et al. (2019)**	Case series	Asian	10	71,1	4	16	30,6				1	3	1	1	ECASS 3			
**Frol et al, 2020**	Case series	Caucasian	19	75	9	9	40,35	7pt:150mg 12pt:110mg	128	0,9	0	4	2	1	ECASS 2			
**Kermer, P., et al. (2017)**	Case series	Caucasian	19	7	11	7	42,3	5pt: 150mg, 14pt: 110mg		0,9		0	1	0				
**Kermer, P., et al. (2020).**	Case series	Caucasian	80	75,9	29	9,7	42,7	32pt:150mg, 48pt: 110mg		0,9		0	3	0				104,5 (63,9)
**Barber, P. A., et al. (2020).**	Case series	Caucasian	51	73,3	14	8					8	2	3					
**Beharry, J., et al. (2020).**	Case series	Caucasian	13	79	4	6					7	2	2	0				80 (57-113)
**Šaňák, D., et al. (2018).**	Case series	Caucasian	13	70	5	7	38,1	8pt: 150, 5pt: 110 mg	427	0,9	0	2	3	1	SITS-MOST			72,3 ±56,1
**Tse, D. M., et al. (2018)**	Case series	Caucasian	6	68	3	21		4 pt 150 mg, 2 pt 110 mg	180	0,9	2	1	1	1	IST-3 modified			
**Küpper, C., et al. (2019).**	Case series	Caucasian	7							0,9	4	0	2	0				
**Agosti, S., et al. (2017)**	Case report	Caucasian	1	71	1	9	29,0	150	210	0,9	0	0	0	0		1,31		
**Alvarez Bravo, G., et al. (2017).**	Case report	Caucasian	1	65	1	19	31,0	150			0	0	0	0				
**Baule, A., et al. (2018).**	Case report	Caucasian	1	89	1	4	38,8	110				0	0	0				
**Berrouschot, J., et al. (2016).**	Case report	Caucasian	1	76	0	11	73,3	110	150		0	0	0	0				
**Binet, Q., et al. (2018)**	Case report	Caucasian	1	55	1	20	37,4	150	120		1	0	0	0		1,18		
**Bissig, D., et al. (2017).**	Case report	Caucasian	1	69	0	12	39,2	150	205	0,9	0	0	0	0		1,12		

**Candelaresi, P., et al. (2020).**	Case report	Caucasian	1	73	1	21		150	115	0,9	1	0	0	0			
**Gawehn., et al. (2016)**	Case report	Caucasian	1	75	0	5	39,0	110	80	0,9	0	0	0	0	1,0		
**Harsha, K. J., et al. (2019)**	Case report	Caucasian	1	74	1	11		150		0,9	0	0	0	0			
**Hieber, M. and J. Bardutzky (2019).**	Case report	Caucasian	1	81	0	6		150	540	0,9	0	0	0	0			
**Hosoki, S., et al. (2018)**	Case report	Asian	1	74	0	6	41,0	110		0,6	0	0	0	0			
**Jala, S. and E. O'Brien (2019).**	Case report	Caucasian	1	77	0	11	51,8	150	93	0,9	0	0	0	0	1,5		
**Kafke, W. and P. Kraft (2016).**	Case report	Caucasian	1	75	1	7	35,5	110		0,9	0	0	0	0	1,1	90	>150
**Lin, Y. T., et al. (2020)**	Case report		1	71	0	9		150	189	0,9	1				1,07		
**Lo, W. T., et al. (2018).**	Case report	Asian	1	78	1	34	50,7	110	115	0,6	0	0	0	0			101
**Loh, C. H. and G. Herkes (2019).**	Case report	Caucasian	1	77	0	8	28,6	110	183	0,6	0	0	0	0	1,1	<35	14
**Maramattom, B. and J. Thomas (2019**	Case report	Caucasian	1	38	0			150	260	0,9	0	0	0	0			
**Meyer, D., et al. (2019).**	Case report	Caucasian	1	73	0	19	27,0		35	0,9	0	0	0	0	1,1		12,5
**Mutzenbach, J. S., et al. (2016).**	Case report	Caucasian	1	68	0	3	34,0	110	100	0,9		0	0	0	<1,2	34,1	
**Ohtani, T., et al. (2019).**	Case report	Asian	1	67	1	7	68,0	110	264	0,9	0	0	0	0			
**Ohya, Y., et al. (2018).**	Case report	Caucasian	1	57	0	22	41,3	110	78	0,9	0	0	0	0			
**Renard, A., et al. (2018).**	Case report	Caucasian	1	74	0	16		110		0,9	1	1	0	0			
**Schäfer, N., et al. (2016).**	Case report	Caucasian	1	67	1	10		150	85	0,9		0	0	0			130
**Schulz, J. G. and B. Kreps (2016)**	Case report	Caucasian	1	76	0	11	72,2	110		0,9		0	0	0			
**Ting, A., et al. (2019).**	Case report	Caucasian	1	68	0	7	47,0	150		0,9	0	0	0	0	1,3	193	16
**Tireli, D., et al. (2017).**	Case report	Caucasian	1	71	0	6	62,0	150	109	0,9		0	0	0	1,3		

**Tsai, L. K., et al. (2018).**	Case report	Asian	1	78	1	24	26,8	110	118	0,9	0	1	1	1			
**Tsai, Y. T., et al. (2018).**	Case report	Asian	1	57	0	9	25,1	110	62	0,9	0	1	0	0			
**Turine, G., et al. (2017).**	Case report	Caucasian	1	85	1	17	32,2	110	298	0,9	0	0	0	0	1,32		112
**von Wowern, F., et al. (2017).**	Case report	Caucasian	1	78	1	11	49,0	150			0	0	0	0			
**Vukorepa, G., et al. (2020)**	Case report	Caucasian	1	80	1	8		110	258	0,9	0	0	0	0			
**Ng et al, (2017)**	Case report	Caucasian	2	46	0	5				0,9	0	0	0	0			
**Pikija et al, (2017)**	Case report	Caucasian	5	88	1	10	71,0	110		0,9		0	0	0		202,4	
				67	0	4	77,0	150		0,9		0	0	0		183,7	
				84	0	10	84,0	150		0,9		0	0	0		31,4	
				85	0	7	36,0	110		0,9		0	0	0		43	
				82	0	18	52,0	110		0,9		0	0	0		172,2	
**Vosko et al, (2017)**	Case report	Caucasian	3	68	0		34,0	110	110	0,9		0	0	0		34	
				84	0	9		110		0,9		0	0	0		79	129
				78	0		134,0	110		0,6		0	0	0			
**Gianndrera et al, (2019)**	Case report	Caucasian	2	78	0	12	47,0	110		0,9	0	0	0	0			
				85	0	22	53,0	110		0,9	1	1	1	1			
**Zhao et al, (2019)**	Case report	Caucasian	3	71	0	3	20,0		101	0,9	0	0	0	0	1		12,8
				85	1	35	31,0		134	0,9	1	0	0	0	1		12
				84	1	14			258	tenecteplase	0	0	0	0			
**Hieber et al, (2018)**	Case report	Caucasian	3	77	0	5		110	45	0,9	0	0	0	0			96,7
				48	0	18		110	75	0,9	1	0	0	0			42,3
				56	0	7		150		0,9	0	0	0	0			53,6
**Cappelari et al. (2017)**	Case report	Caucasian	1	75	1	4		150				0	0	0			
**Facchinetti et al, (2017)**	Case report	Caucasian	1	78	1	4		150				0	0	0			

Table 2: Quality evaluation

Study	Selection	Ascertainment		Causality				Reporting	Overall quality
	Does the patient(s) represent(s) the whole experience of the investigator (centre) or is the selection method unclear to the extent that other patients with similar presentation may not have been reported?	Was the exposure adequately ascertained?	Was the outcome adequately ascertained?	Were other alternative causes that may explain the observation ruled out?	Was there a challenge/rechallenge phenomenon?	Was there a dose–response effect?	Was follow-up long enough for outcomes to occur?	Is the case(s) described with sufficient details to allow other investigators to replicate the research or to allow practitioners make inferences related to their own practice?	
Fang, C. W., et al. (2019)	good	good	good	yes	no	no	yes	yes	good
Frol et al, (2020)	good	good	good	yes	no	no	yes	yes	good
Kermer, P., et al. (2017)	good	good	good	yes	no	no	yes	yes	good
Kermer, P., et al. (2020).	good	good	good	yes	no	no	yes	yes	good
Barber, P. A., et al. (2020).	good	good	good	yes	no	no	yes	yes	good
Beharry, J., et al. (2020).	good	good	good	yes	no	no	yes	yes	good
Šaňák, D., et al. (2018).	good	good	good	yes	no	no	yes	yes	good
Tse, D. M., et al. (2018)	good	good	good	yes	no	no	yes	yes	good
Küpper, C., et al. (2019).	good	good	fair	yes	no	no	yes	yes	fair
Agosti, S., et al. (2017)	good	good	fair	yes	no	no	yes	yes	fair

**Alvarez Bravo, G., et al. (2017).**	good	good	good	yes	no	no	yes	yes	good
**Baule, A., et al. (2018).**	good	good	good	yes	no	no	yes	yes	good
**Berrouschot, J., et al. (2016).**	good	good	good	yes	no	no	yes	yes	good
**Binet, Q., et al. (2018)**	good	good	good	yes	no	no	yes	yes	good
**Bissig, D., et al. (2017).**	good	good	good	yes	no	no	yes	yes	good
**Candelaresi, P., et al. (2020).**	good	good	good	yes	no	no	yes	yes	good
**Gawehn., et al. (2016)**	good	good	good	yes	no	no	yes	yes	good
**Harsha, K. J., et al. (2019)**	good	good	good	yes	no	no	yes	yes	good
**Hieber, M. and J. Bardutzky (2019).**	good	good	good	yes	no	no	yes	yes	good
**Hosoki, S., et al. (2018)**	good	good	good	yes	no	no	yes	yes	good
**Jala, S. and E. O'Brien (2019).**	good	good	good	yes	no	no	yes	yes	good
**Kafke, W. and P. Kraft (2016).**	good	good	good	yes	no	no	yes	yes	good
**Lin, Y. T., et al. (2020)**	good	good	good	yes	no	no	yes	yes	good
**Lo, W. T., et al. (2018).**	good	good	good	yes	no	no	yes	yes	good
**Loh, C. H. and G. Herkes (2019).**	good	good	good	yes	no	no	yes	yes	good
**Maramattom, B. and J. Thomas (2019**	good	good	fair	yes	no	no	yes	yes	fair
**Meyer, D., et al. (2019).**	good	good	good	yes	no	no	good	yes	good
**Mutzenbach, J. S., et al. (2016).**	good	good	good	yes	no	no	good	yes	good
**Ohtani, T., et al. (2019).**	good	good	good	yes	no	no	good	yes	good
**Ohya, Y., et al. (2018).**	good	good	good	yes	no	no	good	yes	good

**Renard, A., et al. (2018).**	good	good	good	yes	no	no	good	yes	good
**Schäfer, N., et al. (2016).**	good	good	fair	yes	no	no	good	yes	fair
**Schulz, J. G. and B. Kreps (2016)**	good	good	good	yes	no	no	good	yes	good
**Ting, A., et al. (2019).**	good	good	good	yes	no	no	good	yes	good
**Tireli, D., et al. (2017).**	good	good	good	yes	no	no	good	yes	good
**Tsai, L. K., et al. (2018).**	good	good	fair	yes	no	no	fair	yes	fair
**Tsai, Y. T., et al. (2018).**	good	good	good	yes	no	no	good	yes	good
**Turine, G., et al. (2017).**	good	good	good	yes	no	no	good	yes	good
**von Wowern, F., et al. (2017).**	good	good	good	yes	no	no	good	yes	good
**Vukorepa, G., et al. (2020)**	good	good	good	yes	no	no	good	yes	good
**Ng et al, (2017)**	good	good	good	yes	no	no	good	yes	good
**Pikija et al, (2017)**	good	good	good	yes	no	no	good	yes	good
**Vosko et al, (2017)**	good	good	fair	yes	no	no	yes	yes	good
**Gianndrera et al, (2019)**	good	good	good	yes	no	no	yes	yes	good
**Zhao et al, (2019)**	good	good	good	yes	no	no	yes	yes	good
**Hieber et al, (2018)**	good	good	good	yes	no	no	yes	yes	good
**Cappelari et al. (2017)**	good	good	good	yes	no	no	yes	yes	good
**Facchinetti et al, (2017)**	good	good	good	yes	no	no	yes	yes	good
**Laxamana et al (2020)**	good	good	good	yes	no	no	yes	yes	good
